# Evidence for anti-inflammatory effects and modulation of neurotransmitter metabolism by *Salvia officinalis* L.

**DOI:** 10.1186/s12906-022-03605-1

**Published:** 2022-05-12

**Authors:** Gemma Margetts, Sotirios Kleidonas, Nawel S. Zaibi, Mohamed S. Zaibi, Kieron D. Edwards

**Affiliations:** 1 The Faculty of Medicine and Health Sciences, The Institute for Biomedical and Bioscience Research, The University of Buckingham, Hunter Street, Buckingham, MK18 1EG UK; 2 Sibelius Ltd, 20 East Central, 127 Olympic Avenue, Milton Park, Abingdon, Oxfordshire OX14 4SA UK; 3 European Genomic Institute for Diabetes (EGID), Hospital Claude Huriez, 59000 Lille, France

**Keywords:** *Salvia officinalis*, Inflammation, Cognitive health, Intestinal cells, Blood brain barrier cells, Monoamines, Dopamine, Serotonin, Gut-brain axis, Phenotypic drug discovery

## Abstract

**Background:**

Cognitive health is of great interest to society, with neuroinflammation and systemic inflammation age-related risk factors that are linked to declines in cognitive performance. Several botanical ingredients have been suggested to have benefits in this area including *Salvia officinalis* (sage), which has shown anti-inflammatory effects and exhibited promising cognitive improvements in multiple human studies. The current study demonstrates anti-inflammatory effects for *S. officinalis* across a broad set of in vitro models in human cells, and adds further evidence to support modulation of acetylcholine and monoamine neurostransmitter levels as mechanisms that contribute towards the benefits of the herb on cognitive health.

**Methods:**

The effect of *S. officinalis* extract on release of multiple cytokines and chemokines was measured in human primary intestinal epithelial cells treated with or without LPS stimulation, and Blood Brain Barrier (BBB) cells in presence or absence of recombinant IL-17A and/or Human IL-17RA/IL-17R Antibody. Antioxidant effects were also assessed in BBB cells incubated with the extract and H_2_O_2_. The anti-inflammatory effects of *S. officinalis* extract were further assessed based on clinically-relevant biomarker readouts across 12 human primary cell-based disease models of the BioMAP Diversity PLUS panel.

**Results:**

*S. officinalis* showed significant attenuation of the release of most cytokines/chemokines into apical media in LPS-stimulated intestinal cells, but small increases in the release of markers including IL-6, IL-8 in basolateral media; where TNF-α was the only marker to be significantly reduced. *S. officinalis* attenuated the release of CRP and VCAM-1 from BBB cells under IL-17A induced conditions, and also decreased H_2_O_2_ induced ROS overproduction in these cells. Phenotypic profiling with the BioMAP Diversity PLUS Panel identified additional anti-inflammatory mediators, and based on a similarity search analysis suggested potential mechanistic similarity to caffeic acid and drugs known to inhibit COMT and MAO activity to modulate monoamine metabolism. Subsequent in vitro assessment showed that *S. officinalis* was able to inhibit the activity of these same enzymes.

**Conclusions:**

*S. officinalis* extract showed anti-inflammatory effects across multiple human cell lines, which could potentially reduce peripheral inflammation and support cognitive health. *S. officinalis* extract also showed the ability to inhibit enzymes related to the metabolism of monoamine neurotransmitters, suggesting possible dopaminergic and serotonergic effects acting alongside proposed cholinergic effects to mediate acute cognitive performance benefits previously demonstrated for the extract.

**Supplementary Information:**

The online version contains supplementary material available at 10.1186/s12906-022-03605-1.

## Background

The average global life expectancy has increased by more than 6 years between 2000 and 2019 and is predicted to continue increasing in a similar way. Global average life expectancy is predicted to reach 77.1 years by 2050 [1]. Many theories about normal aging have suggested that aging is associated with the risk of the decline of neurophysiological functions, which often leads to reductions in cognitive performance and capacity [2]. Consequently, the promotion of successful cognitive health, particularly in elderly populations, is becoming a pressing concern for individuals, society, and public health. The promotion of successful cognitive health involves the prevention of the decline of cognitive function related to neurodegenerative diseases, as well as the enhancement of the brain capacity and cognitive reserve [3].

The role of chronic, low-grade inflammation in increasing the risk of numerous age-related health conditions from type 2 diabetes [4] to cardiovascular disease [5] is well established. There has also been growing awareness of the role that inflammation plays in increasing the risk of cognitive decline [6]. It is widely observed that neurodegenerative diseases including Alzheimer’s disease (AD) [7], Parkinson’s disease (PD) [8], multiple sclerosis (MS), and amyotrophic lateral sclerosis (ALS) [9] are commonly associated with neuroinflammation, which itself is related to systemic inflammation.

Rising from the association of dysbiosis with various cognitive disorders, awareness of the role of the intestinal tract in cognitive and mental health is also growing. There is a growing body of research into the gut-brain axis: A bidirectional communication between gut microbiota and the brain [10, 11]. A key location in the absorption of nutrients from digested food, the inner lining of the small intestine is covered by a single layer of intestinal epithelial cells. Apart from nutrient absorption, the intestinal epithelium plays a variety of critical roles including maintaining barrier integrity, preventing invasion by microbial commensals and pathogens, and modulating the intestinal immune system. Inflammation can damage the intestinal barrier, which enhances the inflammatory response, leading to what is commonly known a leaky gut. Recent data indicate that intestinal inflammation contributes to the pathogenesis of PD [12], and increasing numbers of studies imply that PD may start in the gastrointestinal system years before any motor symptoms develop. Indeed, the dysfunction of the intestinal barrier has been associated with several disease states, including obesity and diabetes [13], cancer [14], and other neurodegenerative diseases [15].

A large number of cytokines, including Interleukin-6 (IL-6), Tumour Necrosis Factor-α (TNF-α), IL-18, IL-1β, and IL-17, are over-expressed in the inflamed gut and have been related to intestinal dysfunction and damage [16]. Such markers may also contribute towards systemic effects of inflammation including neuroinflammation. For example, IL-17A has been highlighted with a role in disruption of the blood-brain barrier (BBB) [17, 18], the protective barrier of the central nervous system (CNS) that separates the blood of the periphery from cerebrospinal fluid (CSF). IL-17A has been shown to induce BBB breakdown by the over-formation of reactive oxygen species (ROS) and inflammatory molecules, and by decreasing the number of tight junctions [18, 19]. The BBB is highly selective and semi-permeable, allowing it to regulate the transport of molecules in and out of the CNS, whilst restricting the entry of pathogens and neurotoxic plasma components. BBB damage and dysfunction can lead to these components leaking into the CNS, and has been associated with several neurodegenerative and autoimmune diseases [20].

Rich in bioactive compounds including phenolic acids and terpenes, *S. officinalis* has been used for decades as a medicinal plant in treating several diseases [21]. Recent studies have shown promising activity in areas such as cancer [22] through to heart disease, dementia, and obesity [23]. In particular, *Salvia* plants have traditional applications in cognitive health dating back hundreds of years [24], and there is a growing body of scientific evidence supporting beneficial effects in this area (reviewed in [25]). This includes several human volunteer studies with extracts of *Salvia* showing cognitive performance benefits in healthy adults [26–30], as well as in AD patients [31].

Our previous investigation of *S. officinalis* extract supported anti-inflammatory effects in adipose cells, as well as attenuation of the cross-talk between peripheral tissues and nerve tissues [32]. The same extract has previously been shown to have cognitive performance benefits in both healthy older and younger adults [28, 30]. In addition to anti-inflammatory effects, modulation of the metabolism of acetylcholine neurotransmitters, by the inhibition of Acetylcholinesterase (AChE), has been proposed as one mechanism by which *Salvia* extracts impart acute cognitive benefits in humans [28]. Among other activities, acetylcholine neurotransmitters play an important role in the encoding of new memories [33], and the inhibition of AChE is a mechanism shared by a number of drugs used in the management of symptoms in AD patients [34].

Monoterpenes, including 1,8-cineole and α-pinene have been proposed as driving the AChE inhibition activity from *Salvia* extracts [29, 35], but phenolic acids such as caffeic and rosmarinic acid have also been shown to have this activity [36, 37]. These phenolic acids have also previously been shown to inhibit the activity of enzymes involved in the metabolism of monoamine neurotransmitters, including Catechol-O-methyltransferase (COMT) and Monoamine Oxidase A (MAO-A) and MAO-B [36]. *S. officinalis* itself has also previously been shown to inhibit MAO-B [38].

The monoamines are a group of neurotransmitters consisting of an amino group connected to an aromatic ring. MAO and COMT enzymes are involved in their breakdown and recycling, which in the case of COMT is limited to the catecholamines subset including dopamine, epinephrine, and norepinephrine. MAO inhibitors are utilised as anti-depressants based upon increasing levels of serotonin in subjects, whereas COMT inhibitors are typically used in combination with L-Dopa, to increase dopamine levels and aid the management of PD symptoms [39].

Serotonin has been demonstrated to have effects on cognitive function (focus and flexibility) and mood [40, 41], whilst dopamine levels are strongly linked to working memory and attention [42, 43]. Indeed, pharmaceutical inhibition of COMT has been shown to increase levels of dopamine in the Pre-Frontal Cortex and have significant effects cognitive performance [44–48]. Furthermore, common genetic variants of COMT and MAO, which alter the stability and/or activity of the enzymes, have been strongly linked to elements of cognitive performance [49] as well as Attention Deficit Hyperactivity Disorder (ADHD) [50–52] and PD [39]. The effect of COMT alleles on executive function and working memory increases with age [53, 54], which may be related to known changes to dopamine signalling with ageing [42]. Similarly, it has been proposed that MAO inhibitors can lead to a reduction in the accumulation of Aβ plaques in Alzheimer’s Disease (AD) through greater monoamine levels [55].

Building on our previous work showing anti-inflammatory effects of *S. officinalis* extract in human cells [32], we now assess these effects across a broader set of cellular contexts. This includes assessment of the extract through in vitro models of the intestinal barrier and BBB, as well as on the BioMAP Diversity PLUS Panel, to gain further insights into the anti-inflammatory activities and other potential mechanisms of action that contribute towards the cognitive performance benefits demonstrated in human volunteers for this extract [28, 30].

## Methods

### Investigational materials

*S. officinalis* extract was provided by Sibelius Ltd. for use in this study. *S. officinalis* plant material of known and invariant provenance was grown in the United Kingdom, according to defined production protocols and Good Agricultural Practice standards. A voucher specimen is available from RHS Garden Wisley herbarium (Surrey, UK; barcode WSY0167384). *S. officinalis* leaves, certified as *S. officinalis* by HPTLC (Alkemist Labs, CA, USA) were dried in an artificially heated (gas fired) hot air drier at temperatures less than 70 °C and then soaked in ethanol (68% w/w) for 48 h. The resultant solution was then concentrated using a climbing film evaporator and dried in a vacuum oven to a final ratio of approximately 7.5:1 dry plant material to extract. The resulting material was milled using a 45 mesh to produce the final Sibelius™: Sage extract, which is a commercial extract standardised to a minimum level of 2.5% w/w rosmarinic acid (batch numbers 43,625/N0587 and 44,878/N0775 used in this study; certificates of analysis in Additional file 1).

### Biochemical analyses

Total phenolic and total tannin levels of the *S. officinalis* extract were measured using the Folin and Ciocalteu method against a tannic acid standard, and total flavonoid levels were measured using the aluminium chloride method against a rutin standard, both based on three technical replicates from the protocols described by Thangaraj et al. [56]. Rosmarinic acid and 1,8-cineole levels were measured by GCMS analysis at RSSL (Reading, UK). Briefly, 2 g of sample added 20 ml of dichloromethane and sonicated for 15 min, of which 2 ml was passed through a syringe filter into a vial. This sample was then run on a GCMS system and quantitated using selected ions against external standards.

### In vitro assessment of anti-inflammatory activity

#### Cell culture

Human primary small intestinal epithelial cells, media and supplements were obtained from Creative Bio-Array, US (cat# CSC-C92295, lot# 1602413) and delivered at a cell density of 0.5 × 10^6^ at passage 3. These cells were isolated from normal human small intestinal tissue. Human small intestinal epithelial cells were cultured in human complete epithelial cell medium (cat#CN-1098X, lot# 1651390). The media was supplemented with 10% fetal bovine serum (FBS; Life Technologies, cat#10500064), Antibiotic-Antimycotic solution (cat# 16B0913), Insulin-Transferrin- Selenium (ITS; #1490), Epidermal growth factor (EGF; #1490), Epithelial cell supplement (cat# C94327) and L-Glutamine (#1413). Cell culture was grown in a T25 coated with 0.1% Gelatin-Based Coating Solution (Sigma UK, cat#SF008) and stored at 37 °C and 5% CO_2_. The cell media was changed after 24 h and then every 48 h until cells were 70–80% confluent. Cells were checked daily and visualised microscopically to check growth progression.

Primary human brain microvascular cells were provided by Innoprot Spain (RefP10361-IM) and have been developed by immortalizing primary human brain endothelial cells with Lenti-SV40 Lentivirus. Cells were delivered at a cell density of 1 × 10^6^ and cultured in Endothelial cell medium (P60104). The media was supplemented with 5% FBS (Gibco, Lot no# 08Q508IK), Antibiotic-Antimycotic solution (Lot no# 28663) and Endothelial cell growth supplement (Lot #26387). Media, FBS and supplements were also sourced from Innoprot, Spain. Cells were grown in a T25 flask and stored at 37 °C and 5% CO_2_. The cell media was changed after 24 h and then every 48 h until the cells were 70–80% confluent. Cells were checked daily and visualised microscopically to check growth progression.

#### LPS cell treatment

Human intestinal cells were treated with a low (5 μg/ml) or high (20 μg/ml) dose of *S. officinalis* extract (batch N0587) in the presence or absence of LPS (*E.coli*; #L4391, Sigma-Aldrich) at 100 ng/ml and incubated for 24 h at 37 °C and 5% CO_2_. Following incubation for 24 h, the medium was removed from both the top (apical) and bottom (basolateral) of the well inserts. The medium was stored at − 20 °C until it was required for cytokine analysis.

#### IL-17A treatment

Human brain cells were treated with *S. officinalis* extract (20 μg/ml; batch N0587) in the presence or absence of recombinant human IL-17A protein (R&D Systems, #7955-IL-025) and/or Human IL-17RA/IL-17R Antibody (R&D Systems, #MAB177–100) and incubated for 24 h at 37 °C and 5% CO_2_. Following incubation for 24 h, the medium was removed and stored at − 20 °C until it was required for cytokine analysis.

#### Measurement of cytokine release

Protein released into the cell culture medium was measured using MSD Cytokines and Chemokines V-PLEX Human assay kits (Meso Scale Discovery, Gaithersburg, MD, USA). Three kits were used: Human Vascular Injury Panel 2 (4-plex) (K15198D), Human Proinflammatory Panel II (4-plex) (K15053D) and Human MCP-1 (K151NND). MSD plates were pre-coated with capture antibodies on independent and well-defined spots. Samples or calibrators (50 μl) were added to each well in duplicate and the plates were sealed and incubated at room temperature and shaken (700–1000 oscillations/min) for 2 h. The plates were washed three times with 200 μl/well of wash buffer and 25 μl of detection antibody solution was added to each well. The plates were then sealed and incubated at room temperature and shaken (700–1000 oscillations/min) for a further 2 h. Finally, the plates were washed by the same method again and 150 μl of reading buffer was added to each plate and read using a Mesoscale Discovery instrument (MSD SECTOR Imager 2400). The unknown levels of cytokines and chemokines released in the cell culture media were determined by the Meso Scale software.

#### Measurement of ROS production

Human brain cells were seeded into 96 well plates and treated *S. officinalis* extract (20 μg/ml) for 24 h at 37 °C and 5% CO_2_. Media was then removed and the cells were incubated with 10 μM of a Fluorescent Substrate (probe) H_2_DCFDA (Millipore, USA, lot no# 3256654) in the dark at 37 °C for 45 min. Cells were washed with 100 μL of phosphate buffered saline (PBS) (Gibco,# 10,010–015) before being treated with 1 mM H_2_O_2_ (Sigma, UK, #216765) for 1 h at 37 °C and 5% CO_2_. Plates were then read using a florescence microplate reader (Spectra MAX Gemini EM, Molecular Devices, California) at Ex/Em 495/519 nm. The probe and H_2_O_2_ were diluted in growth media.

### BioMAP Diversity PLUS

Phenotypic profiling with the BioMAP Diversity PLUS Panel was conducted by Eurofins Discovery (DiscoverX, USA) as described previously [57], with doses of the *S. officinalis* extract (batch N0775) of 2.5 μg/ml, 5 μg/ml, 10 μg/ml, and 20 μg/ml (the previous analysis of the *S. officinalis* extract presented in Additional file 4 was with completed with batch N0587). Human primary cell-based disease models in the BioMAP Diversity PLUS Panel represent a broad set of tissue and disease biology, including: vascular biology (venular endothelial cells) modelled in the Th1 (3C system) and Th2 (4H system) inflammatory environments, as well as in a Th1 inflammatory state specific to arterial smooth muscle cells (CASM3C system); systemic immune responses are modelled in a Th1 inflammation environment (LPS system) or after T cell stimulation (SAg system; both venular endothelial cells and peripheral blood mononuclear cells [PBMCs]); chronic Th1 inflammation driven by macrophage activation (/Mphg system; macrophages and venular endothelial cells) and the T cell-dependent activation of B cells (BT system; PBMCs and B-cells). Lung inflammation is modelled in the Th1 (BE3C system) and Th2 (BF4T system) inflammatory environments (bronchial epithelial cells, or bronchial epithelial cells and dermal fibroblasts respectively); myofibroblast-lung tissue remodelling is assessed in lung fibroblasts (MyoF system); skin biology is addressed in a Th1 cutaneous inflammation environment in keratinocytes and dermal fibroblasts (KF3CT system); and wound healing and tissue remodelling is assessed in dermal fibroblasts (HDF3CGF system). A summary of the cell-based disease models including cell types and stimulation, as well as a full list of the biomarkers assessed in each model is available in Additional file 2. Biomarker activities are determined to be statistically significant, and are thus annotated, when two or more consecutive concentrations of a test agent change in the same direction relative to vehicle controls, are outside of a significance envelope generated from historical control data, and have at least one concentration of test agent with an effect size > 20% (|log_10_ ratio| > 0.1).

### Enzymatic assays

All of the enzymatic assays were conducted in the *S. officinalis* extract batch N0775. The Catechol-O-methyl transferase (COMT) inhibition assay was conducted by Eurofins Discovery (Panlabs, Taiwan), with activity of COMT from porcine liver measured by spectrofluorimetric quantitation of scopoletin based on the method of Müller-Enoch et al. [58]. Monoamine Oxidase-A (MAO-A) and MAO-B inhibition assays were conducted by Eurofins Discovery (Cerep, France). MAO-A activity was measured by photometric detection of 4-OHquinoline using MAO-A enzyme from human placenta based on the method of Weyler and Salach [59], and MAO-B activity was measured by detection of luminescence from methyl ester luciferin using human recombinant MAO-B enzyme based on the method of Tsugeno et al. [60]. The Acetylcholinesterase (AChE) inhibition assay conducted by Eurofins Botanical testing (USA), with spectrophotometric detection of 2-nitro-mercaptobenzoate using AChE enzyme from electric eel based on the method of Vintutha et al. [61].

### Statistical analysis

Statistical analysis and graphical representation of cytokines and ROS data in human primary small intestinal epithelial cells and/or human brain microvascular cells was performed in GraphPad Prism 5 software using one-way ANOVA test flowed by Dunnett’s multiple comparison test.

BioMAP profiles were compared against a proprietary reference database of > 4000 BioMAP profiles of bioactive agents (biologics, approved drugs, chemicals and experimental agents) to classify and identify the most similar profiles. Common biomarker readouts were annotated when the readout for both profiles was outside of the significance envelope with an effect size > 20% in the same direction. Concentrations of test agents that had 3 or more detectable systems with cytotoxicity were excluded from similarity analysis. The similarity between agents was determined using a combinatorial approach that accounts for the characteristics of BioMAP profiles by filtering (Tanimoto metric) and ranking (BioMAP Z-Standard) the Pearson’s correlation coefficient between two profiles. Profiles were identified as having mechanistically relevant similarity if the Pearson’s correlation coefficient was ≥0.7. Briefly, a Pearson’s correlation coefficient (r) was first generated to measure the linear association between two profiles that is based on the similarity in the direction and magnitude of the relationship. Since the Pearson’s correlation can be influenced by the magnitude of any biomarker activity, a per-system weighted average Tanimoto metric was used as a filter to account for underrepresentation of less robust systems. The Tanimoto metric does not consider the amplitude of biomarker activity, but addresses whether the identity and number of readouts are in common on a weighted, per system basis. A real-value Tanimoto metric was calculated first by normalizing each profile to the unit vector (e.g., $A=\frac{A}{\left\Vert A\right\Vert }$) and then applying the following formula: $\frac{A.B}{\left\Vert A\right\Vert +\left\Vert B\right\Vert -A.B}$, where A and B are the 2 profile vectors. Then, it was incorporated into a system weighted-averaged real-value Tanimoto metric in this calculation: = $\frac{\sum Wi. Ti}{\sum Wi}$ . The calculation uses the real-value Tanimoto score for each *i*th system (T_i_) and the weight of each *i*th system (W_i_). W_i_ was calculated for each system in the following formula: $\frac{1}{1+e-100\ast \left( lr-0.09\right)}$, where lr is the largest absolute value of the ratios from the 2 profiles being compared. Based on the optimal performance of reference compounds, profiles were identified as having mechanistically relevant similarity if the Pearson’s correlation coefficient (*r*) ≥ 0.7. Finally, a Fisher r-to-z-transformation was used to calculate a z-score to convert a short tail distribution into a normal distribution as follows: $z=0.5\ {\log}_{10}\frac{1+r}{1-r}$. Then the BioMAP Z-Standard, which adjusts for the number of common readouts (CR), was generated according to the following formula: Z-Standard = z. $\sqrt{CR-3}$. A larger BioMAP Z-Standard value corresponds to a higher confidence level, and this is the metric used to rank similarity results.

## Results

### Anti-inflammatory effects in intestinal epithelial cells

**Fig. 1:**
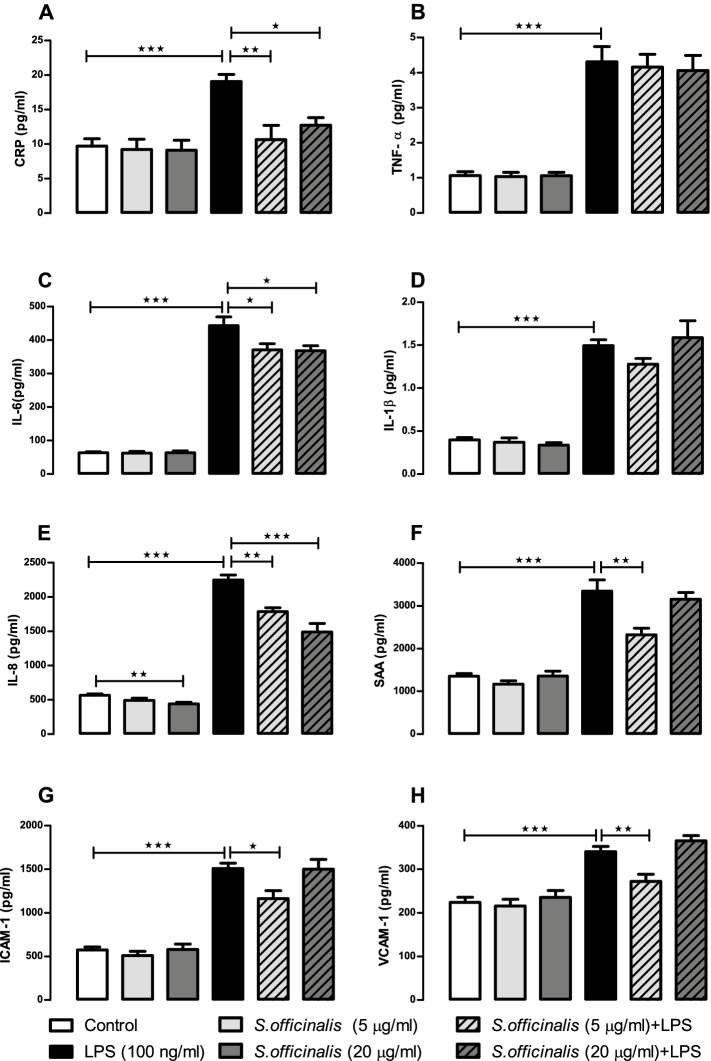
*S. officinalis* extracts attenuation of cytokine and chemokine release by human small intestinal epithelial cells in apical media after 24-h treatment with LPS. Levels of CRP (A), TNF-α (B), IL-6 (C), IL-1β (D), IL-8 (E), SAA (F), ICAM-1 (G) and VCAM-1 (H) released in human small intestinal epithelial apical culture media after 24 h treatment with 5 or 20 μg/mL of *S. officinalis*, and in the presence or absence of 100 ng/ml of LPS. All values are mean ± SEM (*n* = 6, in each treated cell group). See inset keys for treatment identification. Statistical analysis were performed using one-way ANOVA test flowed by Dunnett’s multiple comparison test. Statistical significance is shown as * *p* < 0.05, ** *p* < 0.01 and *** *p* < 0.001

**Fig. 2:**
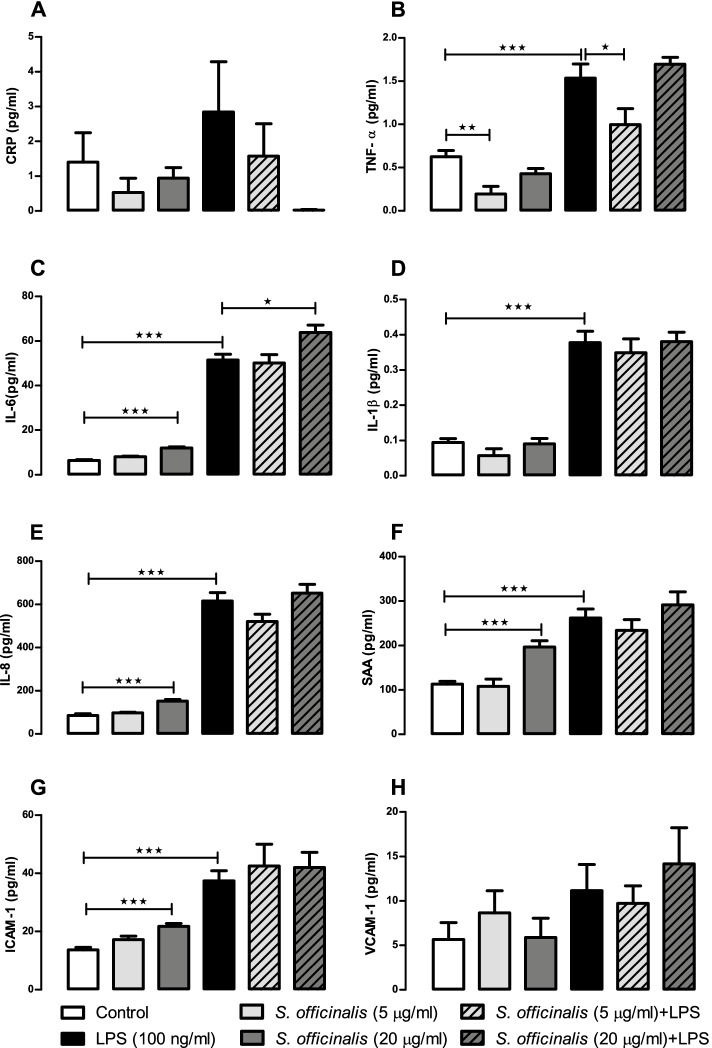
*S. officinalis* extracts attenuation of cytokine and chemokine release by human small intestinal epithelial cells in basolateral media after 24-h treatment with LPS. Levels of CRP (A), TNF-α (B), IL-6 (C), IL-1β (D), IL-8 (E), SAA (F), ICAM-1 (G) and VCAM-1 (H) released in human small intestinal epithelial basolateral culture media after 24 h treatment with 5 or 20 μg/mL of *S. officinalis*, and in the presence or absence of 100 ng/ml of LPS. All values are mean ± SEM (*n* = 6, in each treated cell group). See inset keys for treatment identification. Statistical analysis were performed using one-way ANOVA test flowed by Dunnett’s multiple comparison test. Statistical significance is shown as * *p* < 0.05, ** *p* < 0.01 and *** *p* < 0.001

Given growing understanding of the importance of peripheral inflammation, gut-health and the gut-brain axis on cognitive health, the effects of *S. officinalis* extract were assessed in human small intestinal epithelial cells as a model of the intestinal barrier. Figures 1 and 2 summarise the levels of cytokines and chemokines released into apical and basolateral cell media respectively by human small intestinal epithelial cells incubated with LPS and *S. officinalis* extract for 24 h. Compared to the control group, the LPS treatment caused a significant increase in the release of all eight of the protein markers tested in the apical media (Fig. 1) and all but CRP and VCAM-1 in the basolateral media (Fig. 2), which supports successful induction of pro-inflammatory response by the LPS treatment.

Within the apical media, the 5 μg/ml and 20 μg/ml doses of the *S. officinalis* extract showed significant (*p* < 0.01) reductions in the levels of CRP after stimulation with LPS, returning it close to the basal level of release (44 and 33% reductions respectively; Fig. 1A). The *S. officinalis* treatment also showed a dose-response attenuation of the induction of IL-8 levels in the apical media after LPS stimulation, reducing the release of IL-8 by 20 and 34% for the 5 μg/ml and 20 μg/ml extract doses respectively (Fig. 1E). *S. officinalis* extract also showed a significant decrease (22%) in the basal level of IL-8 release at the 20 μg/ml extract dose treatment (Fig. 1E). Both *S. officinalis* extract doses caused a significant reduction in the release of IL-6 under LPS induced conditions (16 and 17% respectively; Fig. 1C). There was also attenuation of LPS-induced releases for SAA, VCAM-1, and ICAM-1 (31, 20, and 23% reductions respectively) in response to the 5 μg/ml *S. officinalis* extract treatment (Fig. 1F, G and H).

Cytokine and chemokine levels were much lower in the basolateral media than the apical media (Figs. 1 and 2). Within the basolateral media, the 5 μg/ml *S. officinalis* extract dose showed a significant decrease of basal (69%) and LPS-stimulated levels of TNF-α (35%; Fig. 2B). Conversely, *S. officinalis* extract (20 μg/ml) induced a significant increase in the basal level of IL-6 in the basolateral media, as well as after LPS stimulation (Fig. 2C). Similarly, IL-8, SAA, and ICAM-1 basal levels (*p* < 0.001) were significantly increased by the 20 μg/ml treatment dose (Fig. 2E, F, and G).

### Anti-inflammatory effects in human brain microvascular endothelial cells

**Fig. 3:**
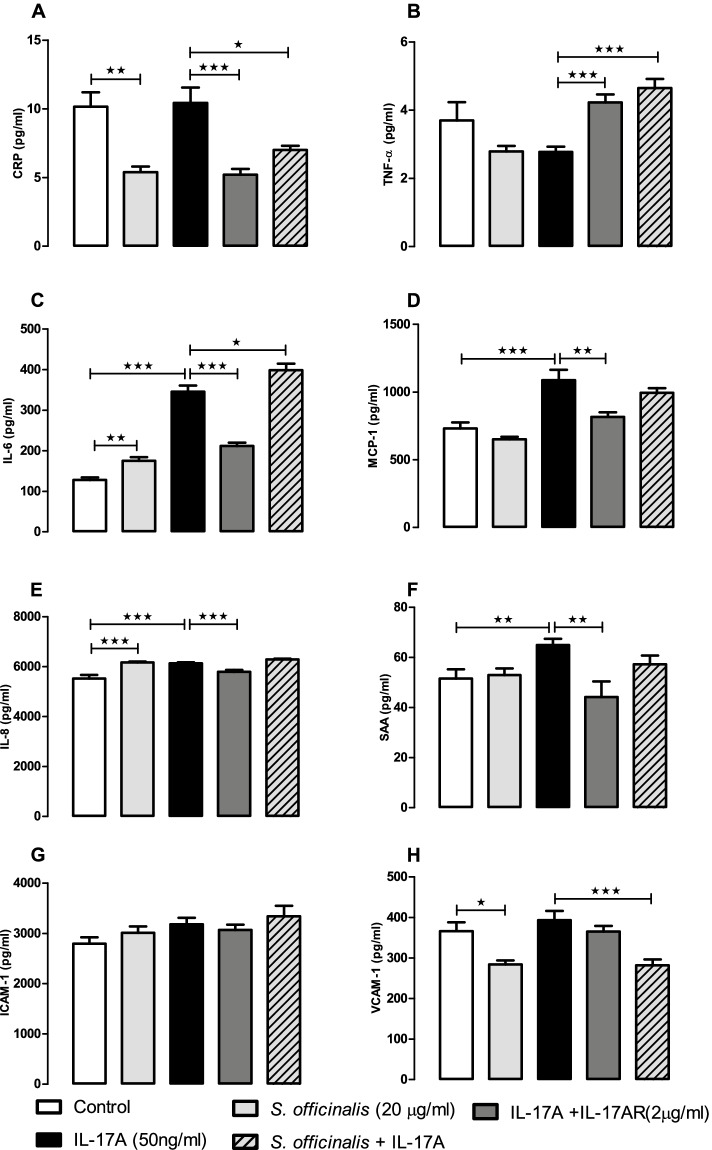
*S. officinalis* extracts attenuation of cytokine and chemokine release by human brain microvascular endothelial cells after 24-h treatment with IL-17A. Levels of CRP (A), TNF-α (B), IL-6 (C), MCP-1 (D), IL-8 (E), SAA (F), ICAM-1 (G) and VCAM-1 (H) released in human brain microvascular endothelial culture media after 24 h treatment with 20 μg/mL of *S. officinalis* or 2 μg/ml IL-17 antibody (IL-17AR), and in the presence or absence of 50 ng/ml of IL-17A. All values are mean ± SEM (*n* = 8, in each treated cell group). See inset keys for treatment identification. Statistical analysis were performed using one-way ANOVA test flowed by Dunnett’s multiple comparison test. Statistical significance is shown as * *p* < 0.05, ** *p* < 0.01 and *** *p* < 0.001

As well as reducing peripheral inflammation, we were interested in investigating the potential anti-inflammatory effects of the *S. officinalis* extract in reducing neuroinflammation. Integrity of the BBB plays an important role in maintaining homeostasis in the brain microenvironment. Inflammation can have negative effects on the function of the BBB, with IL-17 in particular linked to disruption of the BBB [17, 18]. The release of cytokines and chemokines were assessed in human brain microvascular endothelial cells as a model of the BBB. Figure 3 shows the levels of cytokine and chemokine markers released into the media by human brain microvascular endothelial cells either under basal conditions or following incubation with IL-17A for 24 h to induce pro-inflammatory conditions. In both conditions, cells were also either treated with vehicle control or *S. officinalis* extract (20 μg/ml), and in the case of IL-17A induced conditions a third treatment option of IL-17 antibody was also given. Compared to basal conditions IL-17A treatment significantly increased the release of multiple inflammatory markers including MCP-1, SAA, IL-6, and IL-8, supporting induction of inflammation by the treatment (Fig. 3). As might be predicted, IL-17 antibody treatment attenuated the increase in release of all four of these markers (Fig. 3C – F). Although IL-17A treatment did not significantly increase the levels of CRP, both the *S. officinalis* extract and IL-17 antibody caused a significant reduction in the level of CRP in cells under IL-17A induced conditions (respectively by 33 and 35%; Fig. 3A). No significant effect on VCAM-1 was shown by IL-17 antibody and TNF-α showed a significant increase in response to the antibody treatment (Fig. 3B and H).

*S. officinalis* extract showed a significant reduction in the basal release of CRP from human brain microvascular endothelial cells, and as noted above, the extract showed the same attenuating effect on CRP release as IL-17 antibody treatment under IL-17A induced conditions (Fig. 3A). Both treatments also showed an increase in release of TNF-α under IL-17A induced conditions (Fig. 3B). However, the *S. officinalis* extract showed no effect on SAA or MCP-1, and caused slight increases to IL-6 (under both basal and IL-17A induced conditions) and IL-8 levels (basal conditions only) in human brain microvascular endothelial cells, whereas the IL-17 antibody treatment had shown a reduction in these markers (Fig. 3C, D, E and F).

Similar effects of the treatments on CRP and TNF-α suggest that the *S. officinalis* extract is attenuating the IL-17A induction in a similar way to the IL-17 antibody treatment. However, the opposite effect of the *S. officinalis* and IL-17 antibody treatments on levels of IL-6 and IL-8, suggest that there are also differences in the mechanisms that are responsible. The action of independent mechanisms between the treatments is further supported by the *S. officinalis* extract showing significant reduction in the release of VCAM-1 under both basal and IL-17A-induced conditions (22 and 28% reductions respectively), whereas the IL-17 antibody treatment had no significant effect on this marker (Fig. 3D).

**Fig. 4:**
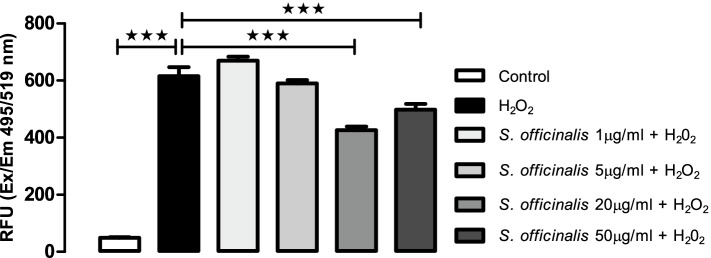
*S. officinalis* extracts attenuation of ROS release by human brain microvascular endothelial cells after 24-h treatment with H_2_O_2_. Levels of ROS released in human brain microvascular endothelial culture media after 24 h treatment with 1, 5, 20 and 50 μg/mL of *S. officinalis* extract and in the presence or absence of 1 mM of H_2_O_2_. All values are mean ± SEM (*n* = 8, in each treated cell group). See inset keys for sage dose. Statistical analysis were performed using one-way ANOVA test flowed by Dunnett’s multiple comparison test. Statistical significance is shown as * *p* < 0.05, ** *p* < 0.01 and *** *p* < 0.001

The induction of ROS formation by IL-17A has been proposed as one mechanism leading to disruption of the BBB caused by this cytokine [18]. *S. officinalis* has previously been shown to have antioxidant activity, linked to high levels of phenolic and other active compounds present in the plant [21]. Therefore, the anti-oxidant activity of the *S. officinalis* extract was also tested in human brain microvascular endothelial cells across a range of doses that had shown anti-inflammatory effects in human cells in the current study and in our previous research [32]. Figure 4 shows the level of ROS production from Human brain microvascular endothelial cells incubated with *S. officinalis* for 24 h. Treatment of the cells with H_2_O_2_ significantly increased ROS production after 24 h compared to the control conditions (Fig. 4). Co-treatment of the cells with *S. officinalis* extract showed a dose-dependent trend in reduction of H_2_O_2_-induced ROS production between the 1 μg/ml and 20 μg/ml extract doses, with the 31% reduction at 20 μg/ml being statistically significant (Fig. 4). The 50 μg/ml *S. officinalis* extract dose also showed a significant reduction in H_2_O_2_-induced ROS production of 19%, but this effect was not as strong as that shown at the lower 20 μg/ml dose (Fig. 4). This suggests a requirement for optimal dosing, which may be related to the complex nature of the botanical extract and possible polypharmacological effects. The data offer further support for anti-oxidant effects of *S. officinalis*, which may contribute to the anti-inflammatory effects observed for the extract in human cells.

### BioMAP Diversity PLUS

**Fig. 5:**
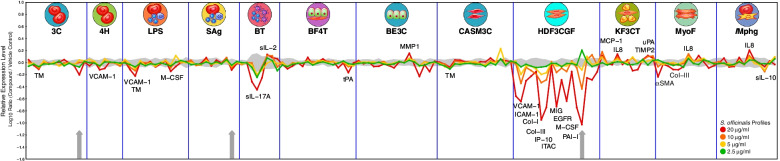
Assessment of *S. officinalis* impact on translational protein biomarkers using the BioMAP Diversity PLUS Panel. Plot showing the biomarker profile of the *S. officinalis* extract. The X-axis shows the quantitative protein-based biomarker readouts across the 12 cell-based disease models (see Methods section for a brief description of each cell system), with log-transformed ratio of the biomarker readouts for the *S. officinalis*-treated sample (*n* = 1) over vehicle controls (*n* ≥ 6) presented on the Y-axis at concentrations of 2.5 μg/ml (green), 5 μg/ml (yellow), 10 μg/ml (orange), and 20 μg/ml (red). The grey region around the Y-axis represents the 95% significance envelope generated from historical vehicle controls. Biomarker activities are annotated in the plot when 2 or more consecutive concentrations change in the same direction relative to vehicle controls, are outside of the significance envelope, and have at least one concentration with an effect size > 20% (|log_10_ ratio| > 0.1). Antiproliferative effects are indicated by a thick grey arrow above the X-axis. A full list of biomarkers assessed in order for each of the 12 cell-based disease models is available in Additional file 2

To further investigate the anti-inflammatory effects of the *S. officinalis* across a broader set of cellular contexts, as well as seek new insights into potential mechanisms of action, the extract was assessed using the BioMAP Diversity PLUS Panel. This systems biology approach provides 148 biomarker readouts across 12 different primary cell-based disease models, advanced analytics, and a comprehensive reference database for insights on mechanism of action, efficacy and safety. The panel is typically applied in pharmaceutical development, but has been used by the U.S. Environmental Protection Agency, and has previously been shown to successfully work for plant extracts [57]. Figure 5 summarises the results of profiling *S. officinalis* extract in BioMAP Diversity PLUS.

The extract showed dose-dependent effects on multiple inflammation and immunomodulatory markers across multiple cell systems. This included reductions in ICAM-1, VCAM-1, IL-17 and IL-10 among other markers (Fig. 5). In the case of VCAM-1 this was reduced across three different cellular disease models. Together with the data from the intestinal epithelial cells and vascular endothelial cells, as well as our previous data [32], this offers strong support for effects on this vascular injury marker by *S. officinalis* extract. Also consistent with our previous results was the reduction in ICAM-1 in the HDF3CGF panel, and mild increases in IL-8 across the KF3CT (keratinocytes and dermal fibroblasts), MyoF (lung fibroblasts) and /Mphg panels (Macrophages and venular endothelial cells; Fig. 5).

The greatest number of annotated activities of the *S. officinalis* extract were shown in the HDF3CGF cell system, which is a model of wound healing and matrix/tissue remodelling comprised of dermal fibroblasts stimulated with TNFα, IL-1β, Interferon-gamma (IFN-ɣ), Epidermal Growth Factor (EGF), basic Fibroblast Growth Factor (bFGF), and Platelet-Derived Growth Factor-BB (PDGF-BB). In addition to the reduction of inflammation markers in this panel, there was also a reduction of Plasminogen activator inhibitor-I (PAI-I), EGF receptor (EGFR), Collagen-I (Col-I) and Col-III, suggesting that the *S. officinalis* extract may also modulate tissue remodelling in response to perturbation (Fig. 5). This cell panel, as well as the 3C (Venular endothelial cells stimulated with TNFα, IL-1β, and IFN-ɣ), and SAg (Venular endothelial cells and peripheral blood mononuclear cells stimulated with TCR ligands) also showed antiproliferative effects (Fig. 5), which may relate to the tissue remodelling effects, but also to potential anti-cancer activities proposed for *S. officinalis* previously [21].

In addition to offering independent support for anti-inflammatory effects, and suggesting potential tissue remodelling and antiproliferative effects for the *S. officinalis* extract, the BioMAP Diversity PLUS Panel also provides the possibility to map a test agent’s response profile against a database of known agents to suggest potential mechanistic similarity with any of these agents. At the top dose tested the *S. officinalis* extract showed above statistically significant (*r* ≥ 0.7) threshold hits to antimicrobial and antifungal treatments, which is perhaps not surprising given traditional application in this area [21] (See Additional file 2). However, perhaps more interesting was the above threshold connection shown with caffeic acid at the 10 μg/ml *S. officinalis* treatment dose, indicating that the *S. officinalis* extract may share some mechanistic similarity with this phenolic acid known to have anti-oxidant, anti-inflammatory and antineoplastic properties [52] (See Additional file 2).

Caffeic acid is synthesized broadly across plant species, and has been shown to have multiple activities including antioxidant and anti-inflammatory [62]. *S. officinalis* is known to contain caffeic acid, and the extract assessed in the current study is relatively rich in rosmarinic acid (4.2% w/w), which is an ester of caffeic acid and 3,4-dihydroxyphenyllactic acid. The level of total phenolic compounds and tannin compounds within the *S. officinalis* extract was estimated by spectrophotometric methods against a Tannic Acid standard at approximately 19% (w/w Tannic Acid Equivalents) and 13% (w/w Tannic Acid Equivalents) respectively (See Additional file 3). Total flavonoid levels were also estimated by spectrophotometry at 17% (w/w Rutin Equivalents; See Additional file 3), which suggests that condensed tannins/proanthocyanadins might make a significant contribution towards the total level of tannins. Together the results support relatively high levels of phenolic compounds within the *S. officinalis* extract, which likely contribute towards the anti-inflammatory and other biological activities of the extract.

The *S. officinalis* extract assessed has previously been demonstrated to provide cognitive performance benefits in older adults as well as younger adults; which appears to be at least partially due to cholinergic properties through the inhibition of acetylcholinesterase (AChE) [28, 30]. It has been proposed that this activity was due to monoterpenes including 1,8-cineole and α-pinene present in high levels in *Salvia* essential oils. These compounds have both been shown to inhibit AChE activity (both with IC_50_ estimates of approximately 0.67 mM) [29, 35]. However, phenolic acids provide alternative candidates to modulate this activity, with caffeic acid (IC_50_ estimate of 23.3 μM [37]) and rosmarinic acid (IC_50_ > 300 μM [36]) having reported inhibition of AChE activity in vitro. Such activity shared between the *S. officinalis* extract and caffeic acid might contribute towards the functional similarity suggested between these entities by the BioMAP Similarity Search analysis.

In addition to cholinergic activities, caffeic acid and rosmarinic acid have also been shown to potentially modulate the metabolism of monoamine neurotransmitters through inhibition of Monoamine Oxidase A (MAO-A; rosmarinic acid IC_50_ 50.1 μM, caffeic acid IC_50_ 138.5 μM [36]), MAO-B (rosmarinic acid IC_50_ 184.6 μM, caffeic acid IC_50_ 247.7 μM [36]), and Catechol-O-methyl transferase (COMT; rosmarinic acid IC_50_ 26.7 μM, caffeic acid IC_50_ 89.9 μM [36]). This is perhaps unsurprising given the structural similarity between caffeic acid and monoamines. Consistent with this, a previous analysis of the *S. officinalis* extract on the BioMAP Diversity PLUS Panel, run across a slightly different concentration range, showed a connection to two COMT inhibitor agents among the top three hits (Entacapone and Phenazopyridine; Additional file 4), although it must be noted that these database hits were below the level of significance (*r* < 0.7) so they cannot be considered as sharing mechanistically relevant similarity based on this analysis alone. However, *S. officinalis* has previously been shown to inhibit MAO-B [38], and in vitro analyses showed that the *S. officinalis* extract was able to inhibit both COMT and MOA-B activity with IC_50_ values of 31.2 μg/ml and 84.2 μg/ml respectively (Table 1; also see Additional file 5). This was in addition to inhibition of AChE activity by the extract (IC_50_ 1794.0 μg/ml; Table 1), supporting the potential for the *S. officinalis* to modulate both acetylcholine and monoamine neurotransmitters.

**Table 1:** Inhibition of enzyme activity by *S. officinalis* extract

Enzyme	IC_**50**_ (μg/ml)
COMT	31.2
MAO-B	84.2
AChE	1794.0

Table summarising the IC_50_ values for *S. officinalis* extract against COMT, MAO-B, and AChE enzymes

Although it is difficult to directly compare doses between different assays, it must be noted that the IC_50_ values for COMT and MAO-B inhibition by the *S. officinalis* extract (31.2 μg/ml and 84.2 μg/ml respectively) were both above the highest concentration tested on the BioMAP platform (20 μg/ml). However, the data suggested that the *S. officinalis* extract could at least partially inhibit the activity of both COMT and MAO-B at doses of 20 μg/ml and below (Additional file 5).

## Discussion

The anti-inflammatory effects of a *S. officinalis* extract have now been demonstrated across multiple cell types and cellular models, supporting results from our previous research [32]. Treatment of human cells with the *S. officinalis* extract caused a reduction in a number of inflammation markers including CRP, TNF-α, SAA, VCAM-1, ICAM-1, MCP-1, IL-17, IL-6, and IL-8, but the data also support an increase in the release of some markers; notably IL-6 and IL-8.

The role of cytokines in maintaining and modulating gut barrier function is complex [63, 64] and difficult to model, especially in an in vitro setting. However, the reduction of the apical release of cytokines in response to the *S. officinalis* treatment in the intestinal epithelial cell model would translate to reduced levels within the intestinal lumen. Given that increased levels of inflammation markers within the intestinal lumen is linked to inflammation [65, 66], a reduction of such markers by the *S. officinalis* extract could contribute towards anti-inflammatory effects and a reduction in peripheral inflammation.

VCAM-1 has shown consistent response to treatment with the *S. officinalis* extract in the current study, being reduced in intestinal epithelial cells and microvascular endothelial cells, as well as in venular endothelial cells – alone and in combination with PBMCs – and dermal fibroblasts. This is in addition to human mature adipocytes from our previous analysis [32]. Given the reported cognitive benefits of *S. officinalis*, it is interesting to note that studies have identified an inverse relationship between VCAM-1 levels and cognitive function in older adults [67, 68]. These studies also showed an inverse relationship between VCAM-1 levels and cerebral blood flow, with atherosclerosis induced by the inflammation marker suggested as a possible cause for this. Reduction in VCAM-1 levels has also been proposed as a mechanism to improve BBB integrity and function, ameliorating age-related neurdogeneration in studies in mice [69].

As noted above, IL-17 has also been linked to BBB function and integrity [17, 18]. The *S. officinalis* extract showed some phenotypic similarity to IL-17 antibody treatment in human microvascular cells, suggesting that the treatment may modulate IL-17A signalling. Such activity may be related to the the pentacyclic triterpene acid ursolic acid, which is known to be rich in *S. officinalis* [70] and has previously been shown to suppress IL-17 production via inhibition of RORɣ [71].

Attenuation of VCAM-1 and IL-17 signalling by the *S. officinalis* extract provides possible mechanisms by which the extract may help to maintain cognitive function through supporting healthy BBB function and improved cerebral blood flow. Healthy BBB function appears to be very important in the prevention or delay of onset for neurodegenerative conditions. For example IL-17 has been shown to play an important role in migration of β-synucelin across the BBB resulting in autoimmune damage to grey matter in a mouse model of MS [72], and defects in BBB function have been suggested as a potential mechanism of increased AD risk associated with the *ApoE4* allele [73]. It is therefore possible that supporting BBB function may have contributed towards the beneficial effects seen in studies of a *S. officinalis* extract in AD patients [31].

Whilst the general pattern has been of a reduction in inflammation markers in response to treatment with the *S. officinalis* extract, the induction of the pro-inflammatory cytokines IL-6 and/or IL-8 has now been observed across several different cellular contexts. Although pro-inflammatory effects cannot be ruled out, IL-6 is known to have pleiotropic effects including anti-inflammatory [74, 75] and IL-8 is also involved in promoting resolution of infections (e.g. phagocytosis, oxidative burst) and angiogenesis, which can help resolve inflammatory stimulus and promote healing [76, 77]. Therefore, the induction of these two cytokines may actually be involved in supporting resolution of inflammation. It is also possible that the increase in IL-6 in human brain microvascular endothelial cells could contribute towards the anti-oxidant effect shown in these cells, based on induction of the stress response transcription factor Nuclear factor-erythroid factor 2-related factor 2 (NRF2) by the cytokine [78]. An NRF2-mediated mechanism might also contribute towards the increase in expression of genes related to NADPH metabolism suggested by previous analysis of gene expression responses to *S. officinalis* extract in human mature adipocytes [32], which would potentially increase glutathione levels and endogenous antioxidant capacity.

Reductions in peripheral- and neuro-inflammation, with associated improvements to BBB function and cerebral blood flow, may contribute long-term benefits towards cognitive health. However, such mechanisms seem less likely to explain the acute cognitive benefits of *S. officinalis* extract, with effects on cognitive performance seen within 1 h of a single dose of the extract [28, 30]. *S. officinalis* has previously been linked to cholinergic effects through inhibition of AChE, with monoterpenes including 1,8-cineole proposed to underlie this effect [29, 35]. Essential oils, which are rich in monoterpenes, account for between 1 and 3% of *S. officinalis* dry weight, and it is therefore very plausible that these compounds contribute to the cognitive enhancing effects observed in studies of *Salvia* essential oils [26, 29, 79]. However, monoterpenes are typically found at much lower levels in solid-liquid extracts of *S. officinalis* [70, 80, 81]. Although 1,8-cineole was not measured in the *S. officinalis* extract batch utilised in the current study, analysis of an equivalent batch only showed 0.4 ppm, which supports very low levels of this monoterpene being present in the *S. officinalis* extract, especially when compared to levels as high as 36.4% w/w in the clinically assessed *Salvia* essential oil [29].

In complement with the monoterpenes, phenolic acids provide very plausible candidates for active compounds in the *S. officinalis* extract. Phenolic acids have been shown to inhibit AChE, COMT, MAO-A and MAO-B [36, 37], which is consistent with such activities shown for *S. officinalis* in the current study and in previous studies [28, 38], and supports modulation of both acetylcholine and monoamine neurotransmitters by *S. officinalis* to impart effects on cognitive performance. Less direct benefits on cognitive health by modulation of neurotransmitters might also be important, such as through the influence of monoamines and catecholamines on the gut-microbiome and gut-brain axis [82].

## Conclusions

The role that chronic- and neuro-inflammation plays in increasing the risk of cognitive decline as well as other health concerns is well established. We have now demonstrated anti-inflammatory effects for a *S. officinalis* extract across multiple cellular models, with notable effects on VCAM-1 and IL-17 signalling. Such effects could translate to reduced neuroinflammation and help to support cognitive health over the longer-term. Our research also adds to the growing body of evidence supporting modulation of neurotransmitter metabolism by *S. officinalis* extract, with increased signalling by acetylcholine and monoamine neurotransmitters likely contributing towards the acute cognitive performance benefits previously shown for *S. officinalis* extract in human volunteers [28, 30].

## Supplementary Information

**Additional file 1.**

**Additional file 2.**

**Additional file 3.**

**Additional file 4.**

**Additional file 5.**

## Data Availability

The datasets used and/or analysed during the current study are available from the corresponding author on reasonable request.

## Abbreviations

AChE: Acetylcholinesterase
AD: Alzheimer’s disease
BBB: Blood brain barrier
COMT: Catechol-O-methyltransferase
CNS: Central nervous system
CRP: C-Reactive Protein
ICAM-1: Intercellular Adhesion Molecule-1
IL-1β: Interleukin-1 Beta
IL-6: Interleukin-6
IL-8: Interleukin-8
IL-17A: Interleukin-17A
LPS: Lipopolysaccharide
MAO: Monoamine Oxidase
MCP-1: Monocyte Chemoattractant Protein-1
PD: Parkinson’s disease
ROS: Reactive Oxygen Species
SAA: Serum Amyloid A
TNF-α: Tumour Necrosis Factor alpha
VCAM-1: Vascular Cell Adhesion Molecule-1
