# Supplementary material for: Evidence for anti-inflammatory effects and modulation of neurotransmitter metabolism by Salvia officinalis L
Source: BMC Complement Med Ther. 2022 May 12;22:131. doi: 10.1186/s12906-022-03605-1 (PMC9101933; doi:10.1186/s12906-022-03605-1)
Supplement: Supplementary file 1 — Additional file 1. [file 12906_2022_3605_MOESM1_ESM.pdf]

SIBELIUS
NATURAL PRODUCTS

CERTIFICATE OF ANALYSIS

Product Name: **SIBELIUS:SAGE**
Description: Dry aqueous ethanolic extract of sage leaf (*Salvia officinalis*)
Batch N^{o}: 43625/N587
Manufacture date: June 2017 Retest date: May 2019
Country of origin: UK

**Parameter**	**Specification**	**Test method**	**Result**
Appearance	Fine green-brown powder	Visual	Complies
Particle size	≥85% passes 355µm sieve (45 mesh)	Internal (QC(P)-8-35)	Complies
Plant:Extract ratio	6.5 – 8.5:1	w/w	Complies
Loss on drying	≤ 7%	AOAC 934.01	4.39%
Total ash content	≤ 10%	AOAC 900.02	3.86%
Rosmarinic acid	≥ 2.5%	Covance Labs., USA	4.11%
Heavy metals:		ICP/MS USP<231>	
Lead	≤ 2ppm		0.053 ppm
Mercury	≤ 0.1ppm		< 0.001 ppm
Arsenic	≤ 1ppm		0.078 ppm
Cadmium	≤ 0.5ppm		0.015 ppm
Total heavy metals	< 10ppm		< 10 ppm
Microbial limits:		USP <2021, 2022>	
Total viable counts (TVC)			
Aerobic bacteria	≤ 10^4		< 10 cfu/g
Yeasts	≤ 10^2		< 10 cfu/g
Moulds	≤ 10^2		< 10 cfu/g
Salmonella spp.	Absent in 10g		Absent in 10g
Escherichia coli	Absent in 10g		Absent in 10g
Staphylococcus aureus	Absent in 10g		Absent in 10g
Pesticide residues	Meets USP <561>	USP <561>	Complies
Residual solvent	Meets USP <467>	USP <467>	EtOH 741 ppm

V1

Sibelius Limited
26 Beaumont Street
Oxford. OX1 2NP. UK
Tel: +44 1865 518910 Fax: +44 1865 511418
www.sibeliusnaturalproducts.com

SIBELIUS
NATURAL PRODUCTS

CERTIFICATE OF ANALYSIS - BOTANICAL			
Supplier Company Name	SIBELIUS LIMITED		
Supplier Address, Contact Information	26 BEAUMONT STREET, OXFORD. OX1 2NP. UK<br>TEL: +44 1865 518910 . EMAIL: steve.caiger@sibeliuslmited.com		
Manufacturing Location	UK		
Name of Manufacturer (if different than Supplier)	SIBELIUS LIMITED<br>Facility: Quest Ingredients Ltd		
Factory Address	Units 1-5, Gooses Foot Industrial Estate, Kingstone. Hereford. HR2 9HY. UK		
**GENERAL INFORMATION**			
Product Name:	SIBELIUS SAGE		Customer Code: SIB4
Grade:	Descriptor or Number: N/A		Manufacturer Date: December 2017
Latin Name:	Salvia officinalis		Testing Date: 19 Jan 2018
Batch Number:	Value: 44878		Expiration/Retest Date: 11/2019
Extraction Solvent:	68% aqueous ethanol		
**ANALYSIS**			
**ITEM DESCRIPTION**	**SPECIFICATION**	**TEST METHOD**	**RESULT**
**PRODUCT CHARACTERISTHICS**			
IDENTIFICATION	MEET STANDARD	HPTLC	COMPLIES
PLANT PART USED	Leaf	HPTLC	COMPLIES
CARRIER USED	N/A	Method / Reference	N/A
PLANT: EXTRACT RATIO	6.5 – 8.5:1	w/w	6.9:1
**PHYSICAL TEST**			
APPEARANCE	Fine green/brown powder	Visual	COMPLIES
COLOR	CHARACTERISTIC	Visual	COMPLIES
AROMA	CHARACTERISTIC	Organoleptic	COMPLIES
TASTE	CHARACTERISTIC / Detail if appropriate	Method / Reference	N/A
PARTICLE SIZE	≥ 85% passes 355µm sieve (45 mesh)	Internal w/w (QC(P)-8-35)	COMPLIES
TOTAL SOLIDS	N/A	Method / Reference	N/A
REFRACTIVE INDEX	N/A	Method / Reference	N/A
VISCOSITY	N/A	Method / Reference	N/A
SOLUBILITY IN WATER	Specify	Method / Reference	N/A
g/cc)	Specify	Method / Reference	N/A
**CHEMICAL TEST**			
ROSMARINIC ACID	Min 2.5%	UPLC	4.2%
MOISTURE	Max 7%	AOAC 934.01	3.67%
ASH	Max 10%	AOAC 900.02	7.04%
TOTAL HEAVY METALS	< 10 ppm	ICP/MS USP<231>	<10 ppm
ARSENIC	Max 1ppm	ICP/MS USP<231>	0.076 ppm
LEAD (AS Pb)	<1 ppm	ICP/MS USP<231>	0.082 ppm
MERCURY	Max 0.1 ppm	ICP/MS USP<231>	0.019 ppm
CADMIUM	Max 0.5 ppm	ICP/MS USP<231>	0.040 ppm
RESIDUAL SOLVENTS	Meet USP <467>	USP <467>	EtOH 1383 ppm
PESTICIDES	Meet USP <561>	USP <561>	COMPLIES
**MICROBIOLOGICAL TEST**			
TOTAL PLATE COUNT	NMT 10^4 cfu/g	USP <2021, 2022>	<10 cfu/g
YEAST & MOULD	NMT 10^2 cfu/g	USP <2021, 2022>	<10 cfu/g
SALMONELLA	ABSENT (cfu/10g)	USP <2021, 2022>	Absent
E. COLI	ABSENT (cfu/10g)	USP <2021, 2022>	Absent
**OTHER OPTIONAL INFORMATION**			

Identity of Authorized Individual for Approval (including Title): Stephen Caiger, Operations Director

Date of Approval: 1 February 2018

26 Beaumont Street Oxford OX1 2NP United Kingdom
T +44(0)1865518910 info@sibeliusnaturalproducts.com
www.sibeliusnaturalproducts.com
Company No. 6949779 VAT No. 114469617
