# Supplementary material for: Evidence for anti-inflammatory effects and modulation of neurotransmitter metabolism by Salvia officinalis L
Source: BMC Complement Med Ther. 2022 May 12;22:131. doi: 10.1186/s12906-022-03605-1 (PMC9101933; doi:10.1186/s12906-022-03605-1)
Supplement: Supplementary file 2 — Additional file 2. [file 12906_2022_3605_MOESM2_ESM.pdf]

3C – Venular endothelial cells stimulated with TNFα, IL-1β, and IFNɣ
Markers: CCL2/MCP−1, CD106/VCAM−1, CD141/Thrombomodulin, CD142/Tissue Factor, CD54/ICAM−1, CD62E/E−Selectin, CD87/uPAR, CXCL8/IL−8, CXCL9/MIG, HLA−DR, Proliferation, SRB

4H – Venular endothelial cells stimulated with IL-4 and Histamine
Markers: CCL2/MCP−1, CCL26/Eotaxin−3, CD106/VCAM−1, CD62P/P−selectin, CD87/uPAR, SRB, VEGFR2

LPS – Venular endothelial cells and PBMCs stimulated with TLR4 ligand
Markers: CCL2/MCP−1, CD106/VCAM−1, CD141/Thrombomodulin, CD142/Tissue Factor, CD40, CD62E/E−Selectin, CD69, CXCL8/IL−8, IL−1alpha, M−CSF, sPGE2, SRB, sTNF−alpha

SAg – Venular endothelial cells and PBMCs stimulated with TCR ligands
Markers: CCL2/MCP−1, CD38, CD40, CD62E/E−Selectin, CD69, CXCL8/IL−8, CXCL9/MIG, PBMC Cytotoxicity, Proliferation, SRB

BT – PBMCs and B cells stimulated with α-IgM and TCR ligands
Markers: B cell Proliferation, PBMC Cytotoxicity , Secreted IgG, sIL−17A, sIL−17F, sIL−2, sIL−6, sTNF−alpha

BF4T – Bronchial epithelial cells and dermal fibroblasts stimulated with IL-4 and TNFα
Markers: CCL2/MCP−1, CCL26/Eotaxin−3, CD106/VCAM−1, CD54/ICAM−1, CD90, CXCL8/IL−8, IL−1alpha, Keratin 8/18, MMP−1, MMP−3, MMP−9, PAI−I, SRB, tPA, uPA

BE3C – Bronchial epithelial cells stimulated with IL-1β, TNFα, and IFNɣ
Markers: CD54/ICAM−1, CD87/uPAR, CXCL10/IP−10, CXCL11/I−TAC, CXCL8/IL−8, CXCL9/MIG, EGFR, HLA−DR, IL−1alpha, Keratin 8/18, MMP−1, MMP−9, PAI−I, SRB, tPA , uPA

CASM3C – Coronary artery smooth muscle cells stimulated with IL-1β, TNFα, and IFNɣ
Markers: CCL2/MCP−1,CD106/VCAM−1, CD141/Thrombomodulin , CD142/Tissue Factor, CD87/uPAR, CXCL8/IL−8, CXCL9/MIG, HLA−DR, IL−6, LDLR, M−CSF, PAI−I, Proliferation, Serum Amyloid A, SRB

HDF3CGF – Dermal fibrobalsts stimulated with IFNɣ, TNFα, IL-1β, EGF, bFGF, and PDGF-BB
Markers: CCL2/MCP−1, CD106/VCAM−1, CD54/ICAM−1, Collagen I, Collagen III, CXCL10/IP−10, CXCL11/I−TAC, CXCL8/IL−8, CXCL9/MIG, EGFR, M−CSF, MMP−1, PAI−I, Proliferation_72hr, SRB, TIMP−1, TIMP−2

KF3CT – Keratinocytes and dermal fibroblasts stimulated with IL-1β, IFNɣ, TGFβ, and TNFα
Markers: CCL2/MCP−1, CD54/ICAM−1, CXCL10/IP−10, CXCL8/IL−8, CXCL9/MIG, IL−1alpha, MMP−9, PAI−I, SRB, TIMP−2, uPA

MyoF – Lung fibroblasts stimulated with TGFβ, and TNFα
Markers: alpha−SM Actin, bFGF, CD106/VCAM−1, Collagen I, Collagen III, Collagen IV, CXCL8/IL−8, Decorin, MMP−1, PAI−I, SRB, TIMP−1

/Mphg – Macrophages and venular endothelial cells stimulated with TLR2 ligand
Markers: CCL2/MCP−1, CCL3/MIP−1alpha, CD106/VCAM−1, CD40, CD62E/E−Selectin, CD69, CXCL8/IL−8, IL−1alpha, M−CSF, sIL−10, SRB, SRB−Mphg

Additional file 2 – Summary of BioMAP® Diversity PLUS® Panel cellular models
Table summarising the cell types and stimulation as well as list of biomarkers assessed in each of the 12 cellular models on the BioMAP® Diversity PLUS® Platform.

S. officinalis extract dose (µg/ml)	Database match	BioMAP Z-standard	Pearson's Score	Mechanism class
20	Chlorquinaldol (1.1µM)	13.990	0.822	Antimicrobial agent
20	Sertaconazole Nitrate (3.3µM)	12.883	0.789	Antifungal agent
20	Butamben (26µM)	11.795	0.837	Anaesthetic agent
10	S31-201 (10µM)	8.992	0.638	STAT3 inhibitor
10	ZM449829 (3.3µM)	8.872	0.632	JAK inhibitor
10	Caffeic acid (28µM)	8.743	0.724	15-Lipoxygense inhibitor
5	Caffeic acid (28µM)	6.701	0.606	15-Lipoxygense inhibitor
5	Coffee extract (450µg/ml)	6.401	0.487	Coffee Extract
5	S31-201 (10µM)	6.117	0.473	STAT3 inhibitor
2.5	Orphenadrine Citrate (3.3µM)	6.886	0.517	Muscle Relaxant
2.5	Ethoxzolamide (30µM)	6.234	0.476	Carbonic Anhydrase Inhibitor
2.5	Acitretin (30µM)	5.845	0.462	Retinoid

Additional file 2 – Table of BioMAP database matches for *S. officinalis* extract
Table summarising the top matching profile hits for the sage extract against the database of treatments run across the BioMAP® Diversity PLUS® platform.
