# Supplementary material for: Evidence for anti-inflammatory effects and modulation of neurotransmitter metabolism by Salvia officinalis L
Source: BMC Complement Med Ther. 2022 May 12;22:131. doi: 10.1186/s12906-022-03605-1 (PMC9101933; doi:10.1186/s12906-022-03605-1)
Supplement: Supplementary file 3 — Additional file 3. [file 12906_2022_3605_MOESM3_ESM.pdf]

Measurement	*S. officinalis* (N0587)	*S. officinalis* (N0775)	Average
Total phenols (w/w% Tannic Acid Equivalents)	18.6	19.8	19.2
Total tannins (w/w% Tannic Acid Equivalents)	12.0	13.5	12.8
Total flavonoids (w/w% Rutin Equivalents)	15.4	18.7	17.1

Additional file 3 – Table of phenolic compound levels in *S. officinalis* extract

Table summarising the levels of total phenols (w/w% Tannic Acid Equivalents), tannins (w/w% Tannic Acid Equivalents), and flavonoids (w/w% Rutin Equivalents) in *S. officinalis* extract batches N0587 and N0775.
