# Supplementary material for: Evidence for anti-inflammatory effects and modulation of neurotransmitter metabolism by Salvia officinalis L
Source: BMC Complement Med Ther. 2022 May 12;22:131. doi: 10.1186/s12906-022-03605-1 (PMC9101933; doi:10.1186/s12906-022-03605-1)
Supplement: Supplementary file 4 — Additional file 4. [file 12906_2022_3605_MOESM4_ESM.pdf]

A

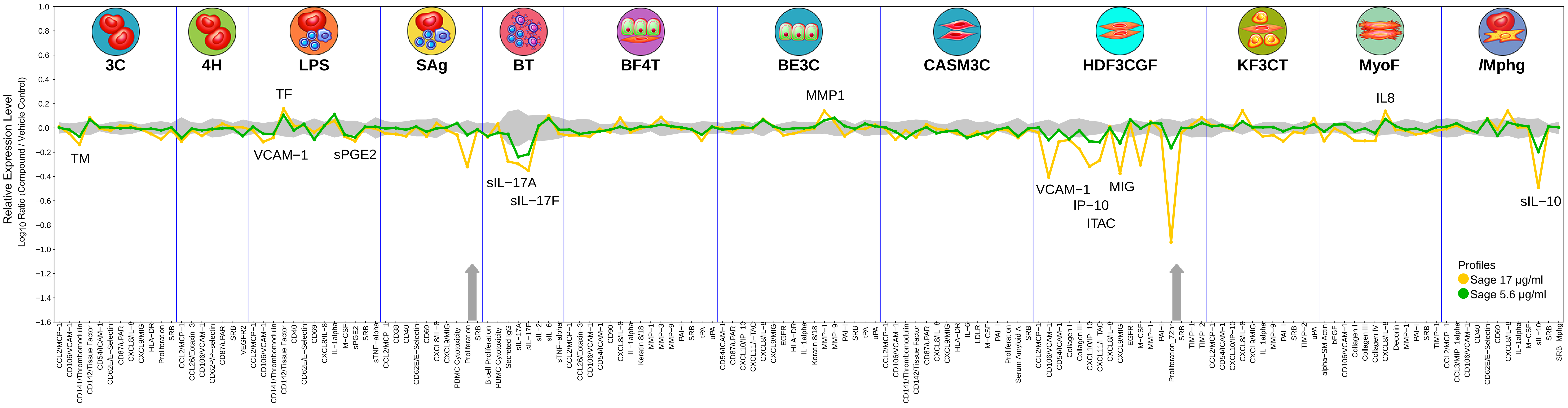

B

Database match	BioMAP Z-standard	Pearson's Score	Mechanism class
Entacapone (30 µM)	7.339	0.544	COMT inhibitor
SR101 (10 µM)	6.862	0.515	RORα/RORɣ Ligand
Phenazopyridine (30 µM)	6.587	0.500	COMT inhibitor

Additional file 3 - BioMAP® Diversity PLUS® Panel analysis of sage extract activity
(**A**) Plot showing the biomarker profile of the sage extract. The X-axis lists the quantitative protein-based biomarker readouts across the 12 cell-based disease models (see Methods section for a brief description of each disease model), with log-transformed ratio of the biomarker readouts for the sage-treated sample (n = 1) over vehicle controls (n ≥ 6) presented on the Y-axis at concentrations of 5.6µg/ml (green), and 17µg/ml (yellow). The grey region around the Y-axis represents the 95% significance envelope generated from historical vehicle controls. Biomarker activities are annotated in the plot when 2 or more consecutive concentrations change in the same direction relative to vehicle controls, are outside of the significance envelope, and have at least one concentration with an effect size > 20% (|log10 ratio| > 0.1). Antiproliferative effects are indicated by a thick grey arrow above the X-axis. (**B**) Table summarising the top matching profile hits for the 5.6µg/ml dose of sage extract against the BioMAP reference Database of test agent profiles.
