# Supplementary material for: Evidence for anti-inflammatory effects and modulation of neurotransmitter metabolism by Salvia officinalis L
Source: BMC Complement Med Ther. 2022 May 12;22:131. doi: 10.1186/s12906-022-03605-1 (PMC9101933; doi:10.1186/s12906-022-03605-1)
Supplement: Supplementary file 5 — Additional file 5. [file 12906_2022_3605_MOESM5_ESM.pdf]

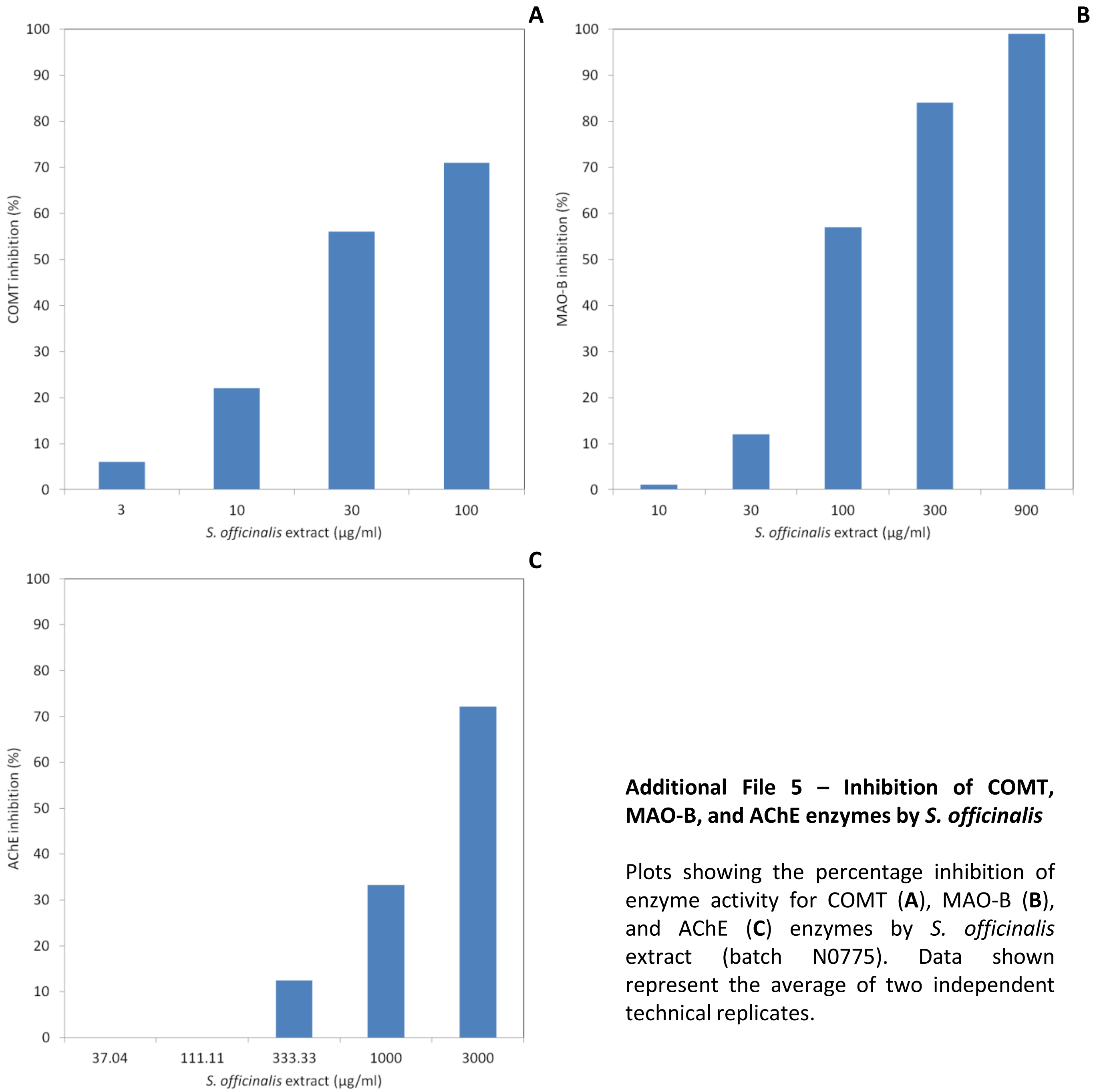

Additional File 5 – Inhibition of COMT, MAO-B, and AChE enzymes by *S. officinalis*

Plots showing the percentage inhibition of enzyme activity for COMT (**A**), MAO-B (**B**), and AChE (**C**) enzymes by *S. officinalis* extract (batch N0775). Data shown represent the average of two independent technical replicates.
